# Long Noncoding RNA LINC01207 Promotes Colon Cancer Cell Proliferation and Invasion by Regulating miR-3125/TRIM22 Axis

**DOI:** 10.1155/2020/1216325

**Published:** 2020-11-22

**Authors:** Ronghong Liu, Wenzeng Zhao, Haigang Wang, Jianbing Wang

**Affiliations:** ^1^Department of Nutrition Section, North China Petroleum Bureau General Hospital, Renqiu 062552, China; ^2^Department of General Surgery, North China Petroleum Bureau General Hospital, Renqiu 062552, China; ^3^Department of Cardiovascular Medicine, North China Petroleum Bureau General Hospital, Renqiu 062552, China

## Abstract

Increasing study has validated that long noncoding RNAs (lncRNAs) are involved in the growth and metastasis of colon cancer. LINC01207 has been reported to play vital roles in certain types of cancer, while the precise function of LINC01207 in the progression of colon cancer remains unclear. The objective of this study was to investigate the effect of LINC01207 on the growth and metastasis of colon cancer cells and to explore the underlying mechanism. We found that the expression of LINC01207 was significantly upregulated in colon adenocarcinoma tissues compared with normal tissues by the GEPIA database. Notably, silencing of LINC01207 significantly suppressed the proliferation, migration, and invasion abilities of SW480 and HT-29 cells. Mechanistically, our data demonstrated that LINC01207 could sponge miR-3125 in colon cancer cells. Moreover, miR-3125 could directly target TRIM22 and negatively regulate its expression. Rescue assays revealed that miR-3125 inhibitor or TRIM22 overexpression significantly reversed the repressive role of LINC01207 knockdown in colon cancer cell proliferation and invasion. In conclusion, LINC01207 exerts an oncogenic role in the progression of colon cancer by absorbing miR-3125 to modulating TRIM22 expression.

## 1. Introduction

Colon cancer is the third most common malignant tumor and the fourth leading cause of cancer-related mortality in the world [1]. Globally, more than one million new cases are diagnosed each year, with approximately 700,000 deaths [2, 3]. Despite significant advances in the diagnosis and treatment of colon cancer over the past two decades, the 5-year survival rate remains below 50%. More than half of colon cancer cases have metastasized to lymph nodes, liver, and lung [4]. Therefore, if we have an in-depth understanding of the mechanism of this disease, we will improve our knowledge of the treatment of this disease and develop novel therapeutic targets.

Long noncoding RNAs (lncRNAs), as a type of noncoding RNA more than 200 nucleotides in length, cannot encode proteins, but play important roles in various physiological and pathological processes. It has been reported that cytoplasmic lncRNAs can regulate the translation, stabilization, and degradation processes of mRNAs and act as competitive endogenous RNAs (ceRNA) to target miRNAs and protein factors to inhibit their activity and facilitate mRNA translation [5]. Additionally, lncRNAs can also interact with RNA, DNA molecules, and protein complexes to exert their functions. Therefore, lncRNAs are reported to function as a chief class of regulatory molecules in various human diseases, including tumorigenesis and cancer development [6, 7]. Some lncRNAs have been shown to be involved in the progression of colon cancer and serve as colon cancer biomarkers, prognostic biomarkers, or potential therapeutic targets [8–10]. LINC01207, located in the genomic 4q32 locus, is reported to be upregulated in lung adenocarcinoma and pancreatic cancer tissues, and its downregulation could inhibit tumor growth and promote apoptosis [11, 12]. Recently, Zeng et al. find that LINC01207 is associated with the prognosis of patients with colorectal adenocarcinoma based on the data from the TCGA database, suggesting that LINC01207 might act as an independent biomarker for colorectal adenocarcinoma [13]. However, the precise role of LINC01207 in the progression of colon cancer remains unclear

In the present study, we are aimed at investigating the biological roles of LINC01207 in the growth and invasion of colon cancer and exploring the underlying mechanism. Our data demonstrated that LINC01207 was upregulated in colon cancer, and the silencing of LINC01207 inhibited colon cancer cell proliferation, migration, and invasion in a ceRNA pattern.

## 2. Materials and Methods

### 2.1. Cell Culture and Transfection

The colon cancer cell line SW480 and HT-29 were purchased from the Cell Bank of the Chinese Academy of Sciences (Shanghai, China). Cells were cultured in RPMI-1640 medium with 10% FBS (Invitrogen, NY, USA) at 37°C. The siRNA targeting LINC01207 (si-LINC01207, 50 nM), miR-3125 mimics (50 nM), miR-3125 inhibitor (50 nM), and corresponding negative controls (50 nM) were obtained from RiboBio (Guangzhou, China). Transfection was performed using Lipofectamine 2000 (Invitrogen) when cells were reached 80% of confluence. The si-LINC01207 sequence was 5′-GCAAATTCCAGAGGTTCAT-3′; the negative control siRNA (NC) sequence was 5′-AAUUCUCCGAACGUGUCACGU-3′. The sequence of miR-3125 mimics was 5′-AGAGAGGUGUCGAAGGAGAU-3′; the sequence of miR-3125 inhibitor was 5′-UCUCUCCACAGCUUCCUCUA-3′.

### 2.2. Quantitative Real-Time Polymerase Chain Reaction (qRT-PCR) Analysis

Total RNA was isolated from colon cancer cells by Trizol reagent (Thermo Fisher Scientific, USA). The miRNA cDNA Synthesis Kit (CWBIO, Beijing, China) or HiFiScript cDNA Synthesis Kit (CWBIO) were used for reverse transcription reaction. Finally, the qRT-PCR analysis was done using miRNA qPCR Assay Kit (CWBIO) or UltraSYBR Mixture Kit (CWBIO). The obtained data were calculated by the 2^−*ΔΔ*Ct^ method. GAPDH or U6 was used as an endogenous control. The relative expression of the target gene was normalized to the negative group. The sequences of primers were as follows: LINC01207, 5′-AAGAACATAGCTGCCCACCC-3′ (sense) and 5′-CGCGAGCTCTGAAGATTTGC-3′ (antisense); TRIM22, 5′-TCTGAGTGGGATGCTGCAAG-3′ (sense) and 5′-AAGCAGGTCGAGAAAAGCGA-3′ (antisense); miR-3125; 5′-TAGAGGAAGCTGTGGAGAGA-3′; GAPDH, 5′-TTCACCACCATGGAGAAGGC-3′ (sense) and 5′-CCACCTGGTGCTCAGTGTAG-3′ (antisense).

### 2.3. CCK8 Assay

Cell proliferation was assessed using CCK8 reagent (Takara, Dalian, China). Following being transfected for 24 h, SW480 and HT-29 cells (1000 cells per well) were seeded in 96-well plates. 10 *μ*l of CCK8 reagent was added into each well at 0, 24, 48, and 72 h, respectively. After incubation for 1.5 h at 37°C, the OD value of each well at 450 nm was measured by microplate reader.

### 2.4. Colony Formation Assay

After 24 h of transfection, cells were digested with 0.25% trypsin and seeded into a 60 mm dish containing 5 ml of medium at a density of 500 cells/dish. Cells were incubated at 37°C for 1-2 weeks, and 1 ml of fresh medium was added in each dish every 2-3 days. SW480 cells were incubated for 10 days and HT-29 cells for 2 weeks, respectively. After incubation was terminated, the supernatant was discarded and the cells were then fixed with 4% paraformaldehyde for 30 min. Following being stained with 0.1% crystal violet for 30 min, the number of colonies was counted.

### 2.5. Transwell Migration and Invasion Assays

Cells (1.0 × 10^5^/ml) were suspended in serum-free medium and added in the upper Transwell chambers precoated with Matrigel (BD Biosciences, NJ, USA) or without precoating. In the lower chamber, 500 *μ*l of medium with 10% FBS was added as a chemoattractant. Following incubation at 37°C overnight, the remaining cells in the upper chamber were wiped offer. The invaded or migrated cells were fixed with 4% paraformaldehyde for 30 min and then stained with 0.1% crystal violet for another 20 min. Penetrating cells were counted and imaged under an optical microscope (Magnification, ×100; Olympus, Tokyo, Japan).

### 2.6. Dual-Luciferase Reporter Assay

Online prediction tools (RegRNA2.0, TargetScan, and miRDB) were performed to predict the corresponding targets in this study. The wild-type sequence of LINC01207 or TRIM22 containing the predicted binding site was cloned into the pmirGLO vector (pmirGLO-LINC01207-wt, pmirGLO-TRIM22-wt), as well as the mutant sequence of LINC01207 or TRIM22 was cloned into the pmirGLO vector (pmirGLO-LINC01207-mut, pmirGLO-TRIM22-mut). The constructed vectors were then cotransfected into colon cancer cells with miR-3125 mimics or miRNA control. After incubation for 48 h, the luciferase activity of each group was measured by a dual-luciferase reporter assay kit (Promega, Madison, WI, USA).

### 2.7. Western Blot Analysis

The proteins were extracted from the cells by RIPA buffer (Beibo, China) and then separated on 10% SDS-PAGE gel and transferred to PVDF membranes (Millipore, USA). The membranes were incubated with blocking buffer (5% no-fat milk in TBST) for 1 h and then cultured with primary antibodies (1 : 1000 dilution; Abcam, USA) at 4°C overnight. After being incubated with HRP-conjugated secondary antibodies (1 : 5000 dilution; Abcam) at room temperature for 1 h, the blots were visualized using the enhanced chemiluminescence detection system (CWBIO).

### 2.8. Statistical Analysis

All the data were presented as means ± SD from more than three independent experiments. The student's *t*-test or one-way ANOVA was used to evaluate the differences between groups. GraphPad Prism 7.0 was used for analysis. *P* < 0.05 was considered to indicate a statistically significant difference.

## 3. Results

### 3.1. Silencing of LINC01207 Impedes the Proliferation of Colon Cancer Cells

**Figure 1:**
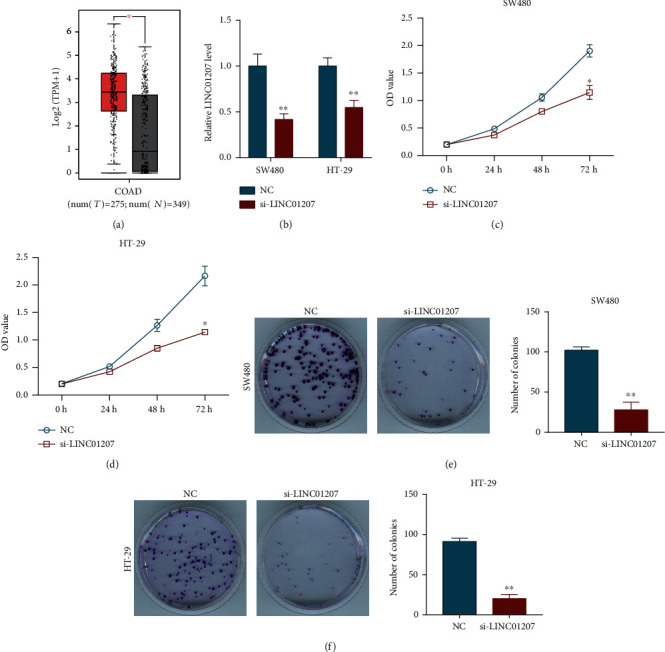
Silencing of LINC01207 suppresses the proliferation of colon cancer cells. (a) Expression of LINC01207 in colon adenocarcinoma (COAD; red box, *n* = 275) tissues and normal tissues (grey box, *n* = 349) by GEPIA database. Expression of LINC01207 in SW480 and HT-29 cells after transfection with si-LINC01207 (50 nM) or si-control (50 nM) (NC) for 24 h. (c, d) CCK8 assay was used to examine the proliferation ability of SW480 (c) and HT-29 (d) cells after indicated transfection. (e, f) Colony-forming ability of SW480 (e) and HT-29 (f) cell was assessed by colony formation assay. Data are the mean ± SD. TPM: transcripts per million. ^∗^*P* < 0.05 and ^∗∗^*P* < 0.01.

From the Gene Expression Profiling Interactive Analysis (GEPIA; http://gepia.cancer-pku.cn/) database [14], we found that LINC01207 was significantly upregulated in colon adenocarcinoma (COAD) tissues compared with normal tissues (Figure 1(a)). Therefore, siRNA targeting LINC01207 was used to silence its expression in SW480 and HT-29 cells (Figure 1(b)). As shown in Figure 1(c), SW480 cell proliferation was greatly inhibited by si-LINC01207. A similar inhibitory effect of si-LINC01207 on HT-29 cell proliferation was also found (Figure 1(d)). Consistently, the silencing of LINC01207 significantly reduced the colony-forming ability of both SW480 and HT-29 cells (Figures 1(e) and 1(f)).

### 3.2. Silencing of LINC01207 Suppresses the Migration and Invasion of Colon Cancer Cells

**Figure 2:**
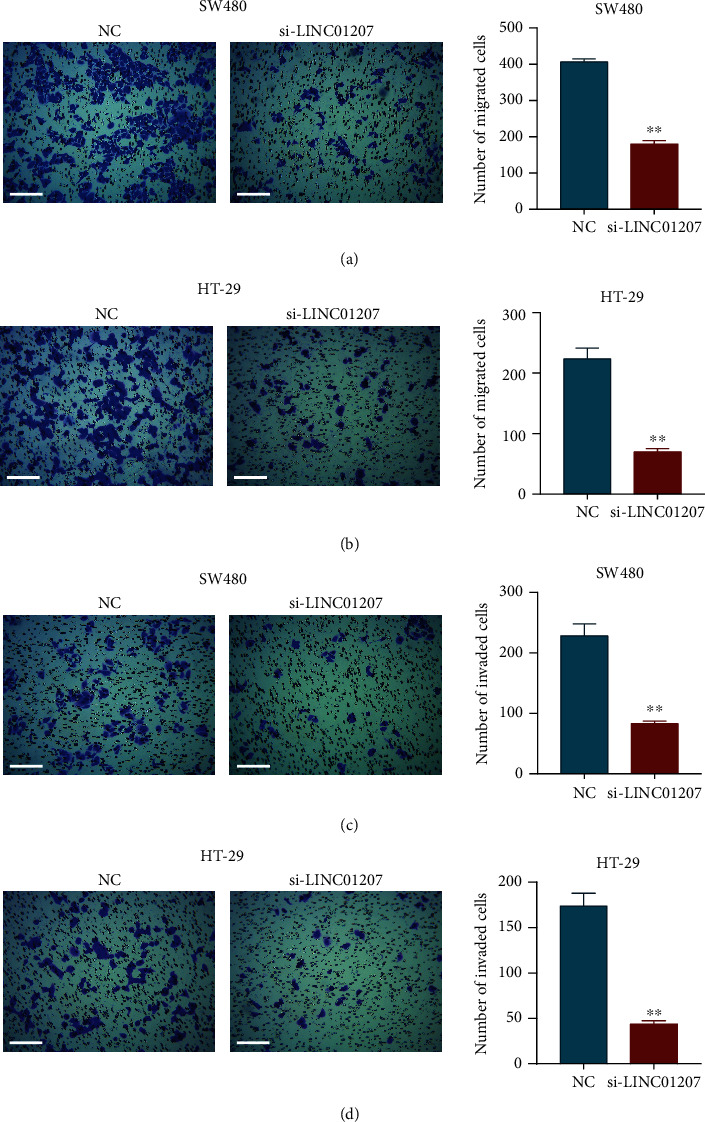
Downregulation of LINC01207 reduces the migration and invasion of colon cancer cells. (a, b) Migration of SW480 (a) and HT-29 (b) cells after silencing LINC01207 was examined using transwell assay. (c, d) Invasion of SW480 (c) and HT-29 (d) cells after indicated transfection was examined using a transwell invasion assay. Scale bar, 200 *μ*M. Data are the mean ± SD. ^∗∗^*P* < 0.01.

Transwell assay was conducted to assess the metastatic ability of colon cancer cells after LINC01207 was silenced. As shown in Figures 2(a) and 2(b), the downregulation of LINC01207 significantly reduced the migration ability of both SW480 and HT-29 cells. A corresponding effect on cell invasion was also observed, which showed a marked decrease in the invasion of SW480 cells transfected with si-LINC01207 compared with the control group (Figure 2(c)). The invasion ability of HT-29 cells was also reduced by the silencing of LINC01207 (Figure 2(d)).

### 3.3. LINC01207 Can Directly Bind to miR-3125 in Colon Cancer Cells

**Figure 3:**
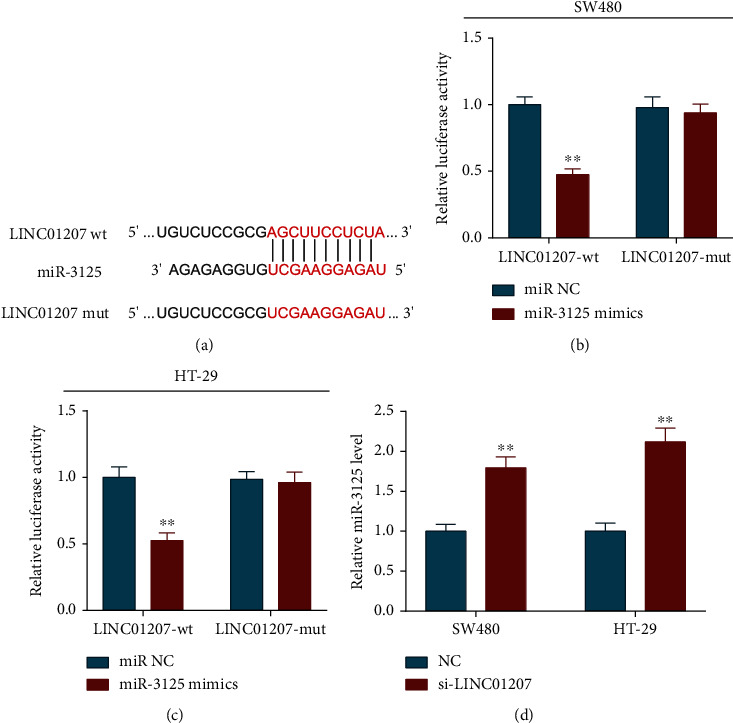
LINC01207 directly and negatively regulates the miR-3125 in colon cancer cells. (a) Putative binding sites between miR-3125 and LINC01207. (b, c) Dual-luciferase reporter assay was performed to examine relative luciferase activity of SW480 (b) and HT-29 (c) cells after cotransfected with pmirGLO-LINC01207-wt or pmirGLO-LINC01207-mut and miR-3125 mimics or miR control (miR NC). (d) Relative expression of miR-3125 in SW480 and HT-29 cells transfected with si-LINC01207. Data are the mean ± SD. ^∗∗^*P* < 0.01.

LINC01207 has been proved to serve as a ceRNA in pancreatic cancer cells [11]. Based on the RegRNA2.0 analysis, we found that miR-3125 had a predicted binding site to LINC01207 (Figure 3(a)). To verify the binding relationship between LINC01207 and miR-3125, the wild-type sequences of LINC01207 or mutant of LINC01207 were cloned into a luciferase reporter vector (pmirGLO-LINC01207-wt/-mut). Dual-luciferase reporter assay showed that miR-3125 mimics markedly reduced the luciferase activity of SW480 and HT-29 cells cotransfected with pmirGLO-LINC01207-wt rather than pmirGLO-LINC01207-mut (Figures 3(b) and 3(c)). Further, the level of miR-3125 was significantly increased in the LINC01207-silenced group compared with the corresponding control group (Figure 3(d)). These results indicate that LINC01207 could directly bind to miR-3125 and negatively regulate its expression in colon cancer cells.

### 3.4. TRIM22 Is a Direct Target of miR-3125 in Colon Cancer Cells

**Figure 4:**
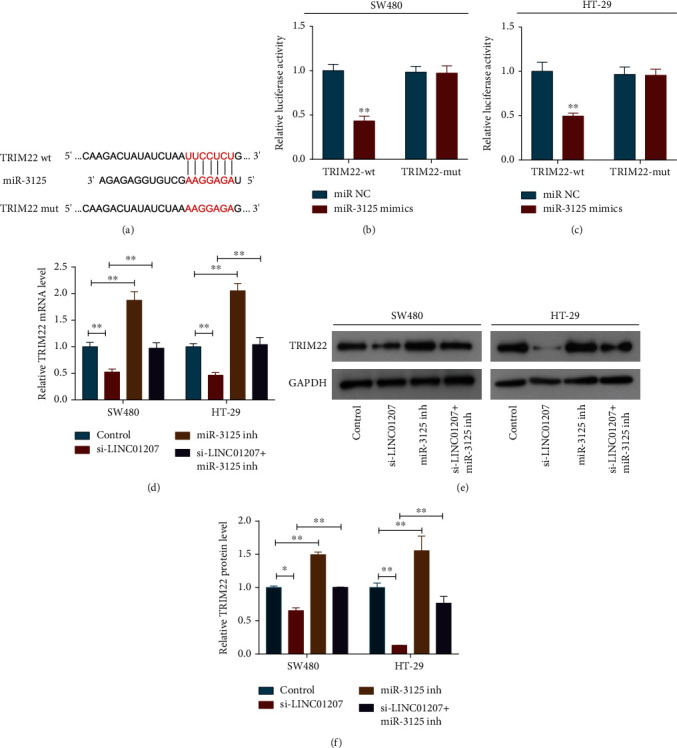
TRIM22 is a direct target of miR-3125 in colon cancer cells. (a) Putative binding sites between TRIM22 and miR-3125 obtained from TargetScan. (b, c) Relative luciferase activity of SW480 (b) and HT-29 (c) cells after cotransfected with pmirGLO-TRIM22-wt or pmirGLO-TRIM22-mut and miR-3125 mimics or miR control. (d) qRT-PCR was used to detect relative expression of TRIM22 mRNA in SW480 and HT-29 cells after indicated transfection. (e) Relative expression of TRIM22 protein in SW480 and HT-29 cells after indicated transfection was detected by western blot analysis. (f) Quantitative analysis of western blot results. miR-3125 inh, miR-3125 inhibitor. Data are the mean ± SD. ^∗^*P* < 0.05 and ^∗∗^*P* < 0.01.

The potential target of miR-3125 was predicted as TRIM22 using TargetScan and miRDB databases (Figure 4(a)). As indicated by dual-luciferase reporter assay, the luciferase activity of TRIM22-wt was remarkably decreased by miR-3125 mimics, but the luciferase activity of TRIM22-mut was not affected (Figures 4(b) and 4(c)). Moreover, we found that TRIM22 expression was upregulated by miR-3125 inhibitor at both mRNA and protein level, but declined in colon cancer cells with the addition of si-LINC01207 (Figures 4(d)–4(f)). Importantly, the decrease in TRIM22 expression caused by si-LINC01207 was offset by miR-3125 inhibitor (Figures 4(d)–4(f)). Collectively, TRIM22 is a target gene of miR-3125 in colon cancer cells, which could be regulated by LINC01207.

### 3.5. LINC01207 Promotes Colon Cancer Cell Proliferation and Invasion by Regulating miR-3125/TRIM22 Axis

**Figure 5:**
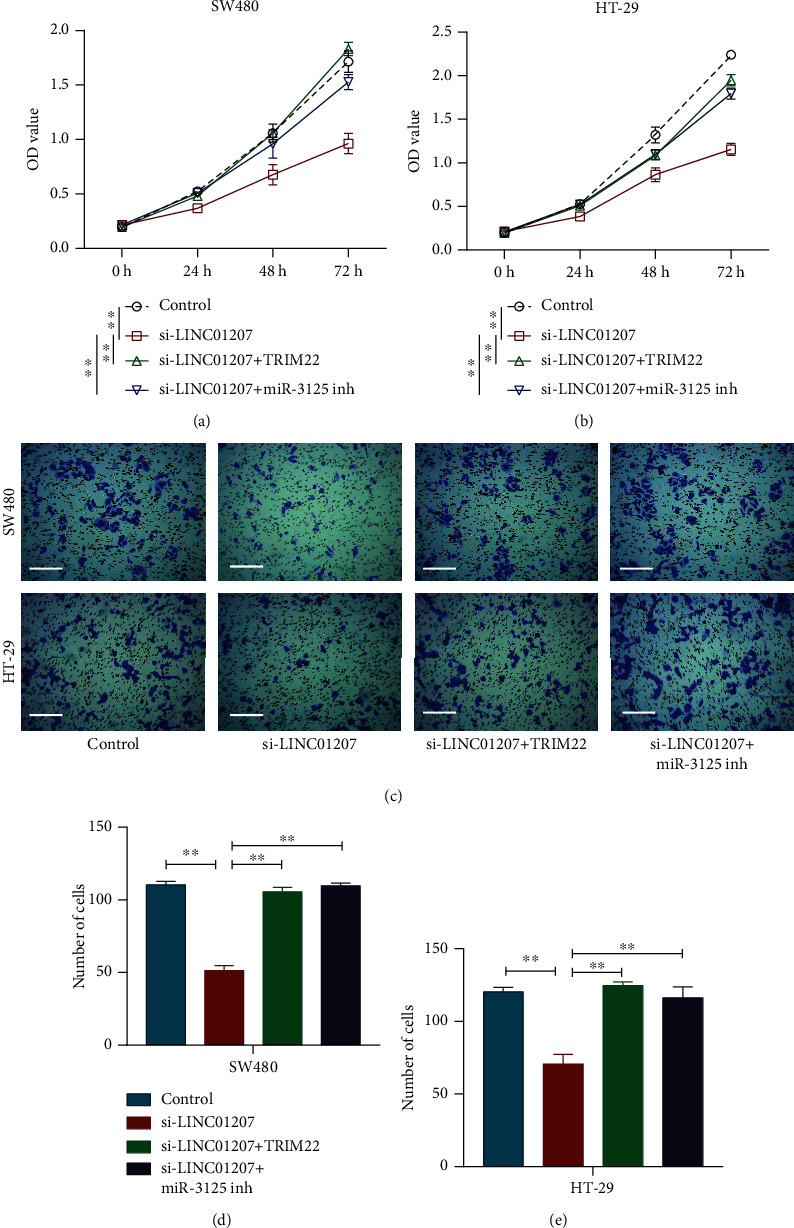
LINC01207 promotes the proliferation and invasion of colon cancer cells by regulating the miR-3125/TRIM22 axis. (a, b) Proliferation of SW480 (a) and HT-29 (b) cells after transfection with si-control, si-LINC01207, or together with miR-3125 inhibitor or pcDNA3.1-TRIM22 was assessed by CCK8 assay. (c) Invasion of SW480 and HT-29 cells with different transfections was determined by transwell assay. miR-3125 inh, miR-3125 inhibitor. (d, e) Quantitative analysis of SW480 (d) and HT-29 (e) cell invasion. miR-3125 inh and miR-3125 inhibitor. Scale bar, 200 *μ*M. Data are the mean ± SD. ^∗∗^*P* < 0.01.

Finally, the rescue assays were performed to further evaluate the effect of LINC01207/miR-3125/TRIM22 axis on the progression of colon cancer. After transfection for 72 h, CCK8 assay revealed that the decrease in the proliferation of SW480 and HT-29 cells caused by LINC01207 knockdown was significantly reversed by either miR-3125 inhibitor or TRIM22 overexpression (Figures 5(a) and 5(b)). Moreover, miR-3125 inhibitor or TRIM22 overexpression significantly reversed the reduced invasion of SW480 cells with LINC01207 knockdown, as well as HT-29 cells (Figures 5(c)–5(e)). These results manifest that LINC01207 promotes the progression of colon cancer by sponging miR-3125 and regulating TRIM22 expression.

## 4. Discussion

Emerging evidence has revealed the critical roles of dysregulated lncRNAs in the growth and metastasis of colon cancer [9, 10]. Herein, we found that LINC01207 was significantly upregulated in colon adenocarcinoma tissues through the GEPIA database. Moreover, silencing of LINC01207 by siRNA interference significantly suppressed the proliferation, migration, and invasion abilities of SW480 and HT-29 cells, highlighting an oncogenic role of LINC01207 in colon cancer. Liu et al. has shown that LINC01207 is highly expressed in pancreatic cancer; its downregulation could induce cell apoptosis and autophagy [11]. In lung adenocarcinoma, high expression of LINC01207 is associated with advanced TNM stage and poor prognosis; its downregulation could inhibit cell proliferation and promote apoptosis [12]. Based on these results, LINC01207 exerts an oncogenic role in cancer progression and might function as a potential therapeutic target for cancer treatment.

LncRNAs have been widely reported to function as ceRNA sponging miRNAs, playing critical roles in the regulation of cancers [15–17]. The lncRNA/miRNA/mRNA network under ceRNA mode further expands miRNA function [17]. Strikingly, LINC01207 has been shown to bind to miR-143-5p in pancreatic cancer cells [11]. Our research demonstrated that miR-3125 was a target of LINC01207 in colon cancer cells; silencing of LINC01207 could upregulate miR-3125 expression in SW480 and HT-29 cells. Hermansen et al. retrospectively analyze 40 samples from patients with glioblastoma by miRNome screening and find that miR-3125 is downregulated in short-term glioblastoma, and its low level is significantly associated with poor prognosis [18]. However, the precise function of miR-3125 in cancer development has not yet been demonstrated, which will be investigated in further study.

Further study was performed to identify the target of LINC01207/miR-3125 in colon cancer cells. Here, we found that TRIM22 was a target of miR-3125. TRIM22 expression was upregulated by miR-3125 inhibitor at both mRNA and protein levels, whereas downregulated by silencing of LINC01207. It is first identified that TRIM22 is an interferon- (IFN-) inducible protein, which plays a role in HIV-1 infection [19]. Additionally, Duan et al. show that TRIM22 may act as a nuclear E3 ubiquitin ligase [20]. Emerging evidence has shown that TRIM22 plays critical roles in tumorigenesis and cancer development, including chronic myeloid leukemia [21], nonsmall cell lung cancer [22], and gliomas [23]. Obad et al. reveal that TRIM22 is a direct target gene for p53, which could be upregulated by p53 and inhibit leukemia U937 cell proliferation and differentiation, suggesting a tumor-suppressive role of TRIM22 in leukemia [24]. However, subsequent studies confirm that the silencing of TRIM22 decreases chronic myeloid leukemia K562 cell proliferation and invasion and triggers cell cycle arrest and apoptosis, indicating an oncogenic role of TRIM22 [21]. Liu et al. show that TRIM22 is upregulated in nonsmall cell lung cancer tissues; its high expression is associated with poor prognosis [22]. TRIM22 overexpression could enhance A549 cell proliferation, invasion, cell cycle progression, and epithelial to mesenchymal transition (EMT) by activating the Akt/GSK3*β*/*β*-catenin signaling pathway [10]. In glioma, TRIM22 is also found to be upregulated, and its downregulation could inhibit tumor growth, invasion, and the Wnt/*β*-catenin signaling pathway [23]. These findings reveal dual roles of TRIM22 in tumor progression, which may be attributed to differences in organ specificity and cellular environment. In the current study, our data demonstrated that as the target gene of miR-3125, TRIM22 overexpression significantly reversed the inhibitory effects of LINC01207 knockdown on SW480 and HT-29 cell proliferation and invasion. These results indicate that TRIM22 may exert an oncogenic role in colon cancer, which needs to be further studied in the future. Additionally, we found that the miR-3125 inhibitor also reversed the decreased cell proliferation and invasion caused by the silencing of LINC01207. Therefore, we speculate that LINC01207 exerts an oncogenic role in the development of colon cancer by regulating the miR-3125/TRIM22 axis.

In sum, our data demonstrated that the silencing of LINC01207 inhibited cell proliferation and invasion in colon cancer by regulating the miR-3125/TRIM22 axis, indicating an oncogenic role of LINC01207. These findings suggest that LINC01207 may function as a novel therapeutic target for colon cancer treatment.

## Data Availability

The data used to support the findings of this study are available from the corresponding author upon request.

## Abbreviations

ceRNA: Competing endogenous RNA
GEPIA: Gene expression profiling interactive analysis
lncRNA: Long noncoding RNA.
